# Mediating factors in the relationship between smartphone addiction and sleep quality: depression, stress, and anxiety

**DOI:** 10.1590/1806-9282.20242069

**Published:** 2025-08-08

**Authors:** Nurten Elkin, Hasan Kütük, Ayşe Mücella Soydan, Sultan Çakmak Tanrıver

**Affiliations:** 1 Istanbul Gelisim University, Faculty of Health Sciences – İstanbul, Türkiye.; 2 Yildiz Technical University, Faculty of Education, Department of Psychological Counseling – İstanbul, Türkiye.; 3 Istanbul Gelisim University, Faculty of Health Sciences, Department of Nursing – İstanbul, Türkiye.

**Keywords:** Smartphone addiction, Depression, Stress, Anxiety, Sleep quality

## Abstract

**OBJECTIVE:**

The aim of the study was to examine the mediating role of depression, stress, and anxiety in the relationship between smartphone addiction and sleep quality.

**METHODS:**

Data were collected from 514 university students using validated self-report measures. Correlation and mediation analyses were performed to examine the relationships between variables.

**FINDINGS:**

Smartphone addiction is negatively associated with sleep quality and positively related to depression, stress, and anxiety. Depression, stress, and anxiety are negatively correlated with sleep quality. The analysis revealed that these three factors partially mediate the relationship between smartphone addiction and sleep quality.

**CONCLUSION:**

The findings indicate that interventions targeting smartphone addiction could potentially alleviate its adverse effects on sleep quality, particularly by addressing underlying psychological factors such as depression, stress, and anxiety. These results obtained during the research process were discussed in relation to similar studies in the literature.

## INTRODUCTION

The university period is a period in which individuals experience a difficult process with their efforts to gain identity and independence and try to adapt to personal and social changes^1^. During this process, distancing from family and coping with the problems brought by university life can lead to various psychological and behavioral problems in individuals, such as introversion, irritable outbursts, alcohol and cigarette use, addictions, academic anxiety, suicide attempts, and family conflicts^2^. Today, it is known that university students frequently use their smartphones; while turning to these devices for digital socialization, they may encounter problems such as a lack of communication within the family and disconnection from real life^3^. Excessive use of smartphones, whose use is rapidly increasing, negatively affects health. Neck pain, sleep disorders, depression, and chronic stress are some of them^4^. Continued smartphone use despite awareness of health risks can be classified as technological addiction, specifically smartphone addiction. The factors defined for smartphone addiction are that the phones are always on, they are used continuously, and their use causes social and financial difficulties.

It is well-established that melatonin levels fluctuate during adolescence, and this alteration may have an impact on sleep quality^5^. Similarly, it is stated that lower sleep quality is observed due to vasomotor effects after menopause^6^. Literature shows that smartphone addiction is associated with sleep quality^4^. In studies conducted on adolescents and university students, significant relationships have been found that smartphone addiction or smartphone overuse negatively affects sleep quality^7^. This situation can also increase students’ levels of depression, anxiety, and stress, and thus cause them to be physically asocial and inactive^8^.

This study contributes to the existing literature by addressing psychological variables that may mediate the relationship between smartphone addiction and sleep quality among university students. Unlike previous studies that have primarily focused on direct relationships, this research highlights the mediating roles of depression, anxiety, and stress, offering a more comprehensive understanding of the pathways through which smartphone addiction may impair sleep quality. The findings of this study are expected to inform intervention strategies aimed at promoting healthier smartphone use and improving psychological well-being and sleep quality among young adults.

Hypotheses of the study:

H1: There is a significant relationship between smartphone addiction and sleep quality, between smartphone addiction and depression, anxiety, and stress, and between sleep quality and depression, anxiety, and stress in university students.

H2: The smartphone addiction variable is a negative predictor of the sleep quality variable.

H3: Depression, anxiety, and stress variables have a mediating role in the effect of the smartphone addiction variable on the sleep quality variable.

## METHODS

This research is a descriptive study that examines the mediating role of depression, anxiety, and stress in the relationship between smartphone addiction and sleep quality among university students, utilizing a relational screening model. In the model established within the scope of this research, the predictor variable was planned as smartphone addiction (X), the predicted variable as sleep quality (Y), and the mediating variables as depression (M1), anxiety (M2), and stress (M3).

### Participants

The sample of this study consists of university students studying in different departments. A total of 514 individuals, 396 (77%) female and 118 (23%) male, participated in the study. The age range of the sample group varied between 18 and 45 years (M_age_=21.82; SD=3.28). The convenience sampling method was preferred in the study. In this type of sampling, data is collected by applying the application to individuals encountered by the practitioner at certain times. It is a method used especially in studies where the universe is very large.

### Measures

The Sleep Hygiene Index aims to evaluate environmental and behavioral variables that may affect sleep quality. The scale was developed by Mastin et al^9^. It is a 13-item scale that evaluates sleep quality. It can be said that as the scores that can be obtained from the scale increase, sleep quality decreases. Cronbach's alpha reliability coefficient regarding the reliability values of the measurement tools was calculated as 0.75.

The Depression Anxiety Stress Scale-21 (DASS-21) was developed by Henry and Crawford^10^. The scale, consisting of 21 items, is a 4-point Likert-type. The DASS-21 is a set of three subscales with seven items in each subscale: depression, anxiety, and stress. It can be said that as the score obtained from the scale increases, the level of psychological distress also increases. Cronbach's alpha reliability coefficient regarding the reliability values of the measurement tools was calculated as 0.93.

The Smartphone Addiction Scale (SAS) was developed by Kwon et al^11^. It is a 10-item scale that evaluates the risk of smartphone addiction. Items are on a 6-point Likert scale ranging from 1 to 6. Higher scores indicate a higher risk of smartphone addiction. Cronbach's alpha reliability coefficient regarding the reliability values of the measurement tools was calculated as 0.93.

### Procedure

The data were collected from university students continuing their education in 2023. Informed consent was obtained from each participant before participating in the study. All stages of the study were meticulously conducted with the ethical approval of Gelisim University Ethics Committee with the number 2023-06-93, taking into account the Declaration of Helsinki.

### Statistical analysis

The steps listed below were followed while analyzing the data. Reliability, descriptive statistics, and correlational analyses of the data in the sample group were carried out through IBM SPSS Statistics 24. Since the skewness and kurtosis coefficients were in the range of +1.5 and −1.5, it was decided that the data were normally distributed. Then, the correlational relationships between smartphone addiction, sleep quality, depression, anxiety, and stress levels of university students were examined. Conditional process analysis was conducted to test the hypotheses of the study. Hayes defined this type of analysis as a mediation model based on regression analysis in which moderators can also be included^12^. When the literature was examined, it was seen that this type of analysis is explained as regression-based mediation analysis used to test theoretical models^13^.

## FINDINGS

First of all, the analyses related to the first hypothesis of the study were conducted. Since the assumption of normal distribution was met, the correlation coefficients between the variables were analyzed, and the results are presented in Table 1.

**Table 1:** Bilateral relationships between smartphone addiction, sleep quality, depression, anxiety, and stress.

	Smartphone addiction	Sleep quality	Depression	Anxiety
Smartphone addiction	–			
Sleep quality	-0.40	–		
Depression	0.30	-0.42	–	
Anxiety	0.38	-0.44	0.58	–
Stress	0.33	-0.45	0.68	0.63

p≤0.01

When Table 1 was examined, it was seen that there was a negative relationship between smartphone addiction and sleep quality (r=-0.40), and a positive relationship between depression (r=0.30), anxiety (r=0.38), and stress (r=0.33). There was also a negative relationship between sleep quality and depression (r=-0.42), sleep quality and anxiety (r=-0.44), and sleep quality and stress (r=-0.45). Based on these findings, the first hypothesis tested was accepted, and these findings were consistent with the results in the literature.

When depression, anxiety, and stress, which are considered as mediating variables in the conditional process analysis based on Baron and Kenny's three-stage assumption, are included in the model, a significant decrease or disappearance of significance is expected in the relationship between smartphone addiction and sleep quality^14^. If there is a significant decrease, depression, anxiety, and stress will have a partial mediating role, while if the significance disappears, depression, anxiety, and stress will have a full mediating role. After this analysis, the significance of the mediating variable is analyzed with the bootstrap test. The bootstrap test determines the significance level of the indirect effect^15^. In this study, the bootstrap value was increased to 5,000 in order to determine the significance of mediation in this study.

### Mediation analysis

**Figure 1:**
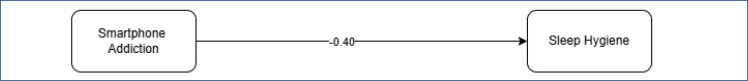
Model of the smartphone addiction variable for predicting sleep quality.

In order to test the mediation model, there should be a significant relationship between smartphone addiction and sleep quality. In line with this, analyses related to the second hypothesis of the study were conducted. The findings of the analyses are given in Figure 1.

According to the findings, smartphone addiction is a negative predictor of sleep quality. According to this regression model, smartphone addiction explains 16% of the total variance [r^2^=0.16; F(1,512)=102.80; p≤0.01]. Therefore, the first assumption for the mediation test was met, and the second hypothesis of the study was accepted.

**Figure 2:**
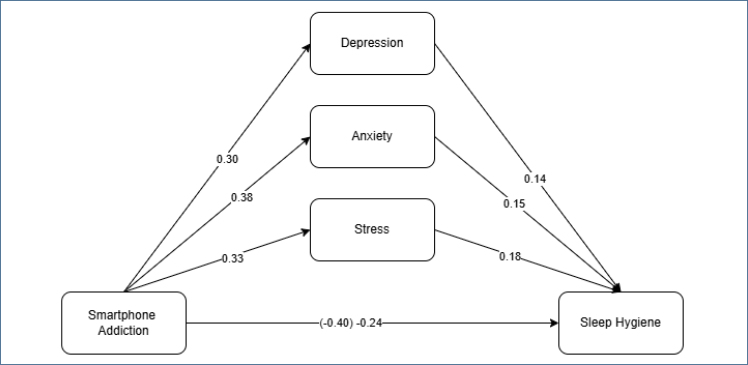
The mediating role of depression, anxiety, and stress in the relationship between smartphone addiction and sleep quality.

When depression (M1), anxiety (M2), and stress (M3) are added to the relationship between smartphone addiction (X) and sleep quality (Y) as a mediating variable in the analysis process related to the third and final hypothesis of the study, it is expected that the effect of smartphone addiction on sleep quality will decrease or disappear completely. Therefore, the last hypothesis of the study was tested. The results obtained are given in Figure 2.

The coefficient of the relationship between smartphone addiction and sleep quality was found to be −0.40 (β=-0.40; p≤0.05). When depression, anxiety, and stress were added to the relationship between these two variables as mediating variables, the relationship coefficient changed to −0.24 (β=-0.40; p≤0.05). The fact that the relationship coefficient changed by 0.16 and maintained statistical significance indicates that depression, anxiety, and stress have a partial mediating role (c′=0.16; p≤0.01). This finding confirmed that depression, anxiety, and stress have a partial mediating role in the effect of smartphone addiction on sleep quality, and the final hypothesis of the study was accepted. A bootstrap test was conducted to determine the significance of the partial mediating role of depression, anxiety, and stress. According to the results obtained from the bootstrap test, it was determined that there was no zero (0) value between the lower and upper limits in the 95% confidence interval for depression (95%CI −0.07 to −0.01), anxiety (95%CI −0.10 to −0.01), and stress (95%CI −0.10 to −0.01) variables. This shows that the result of the bootstrap test is significant, meaning that depression, anxiety, and stress have a partial mediating role in the relationship between smartphone addiction and sleep quality.

## DISCUSSION

The first finding of the analysis revealed that there is a negative relationship between smartphone addiction and sleep quality. University students may encounter difficulties such as disruption in sleep patterns, late-night habits, and decreased sleep quality due to environmental changes, constant messaging, and social media monitoring^16^. Upon reviewing existing studies, it has been determined that smartphone addiction is associated with sleep quality, with a significantly higher frequency of poor sleep quality observed among students with smartphone addiction compared to those without it. Therefore, smartphone addiction is identified as one of the risk factors for poor sleep quality^17,18^.

This study found a positive relationship between smartphone addiction and depression, anxiety, and stress. Elhai et al. stated that depressed and anxious individuals use smartphones more frequently for news and entertainment purposes^8^. Smartphone addicts are more likely to have sleep disorders than less addicted users, which may be a risk factor for depression. Excessive smartphone use leads to attention problems and stress. Sarhan's study with 367 university students showed that smartphone addiction is positively associated with depression, anxiety, and stress. These studies have shown that depression, anxiety, and stress are significantly and positively associated with smartphone addiction^19^.

Additionally, this study has also determined a negative relationship between sleep quality and depression, anxiety, and stress. Individuals with depression experience problems such as difficulty falling asleep and staying asleep due to disruption of sleep patterns^20^. Many individuals with depression also experience an increase in anxiety and stress levels. As a result of the studies, it was determined that there was a statistically negative relationship between sleep quality and depression, anxiety, and stress and that sleep quality was low in students with high levels of depression, anxiety, and stress^21^. This study showed that depression, anxiety, and stress have a partial mediating role in the relationship between smartphone addiction and sleep quality. When the studies were examined, Demirci et al. stated that there was a positive correlation between SAS scores and depression, anxiety, and poor sleep quality in 319 university students and that they could be independent predictors of smartphone addiction^22^. In Choksi's study, it was observed that there was a significant positive relationship between stress, anxiety, depression, and sleep quality and smartphone addiction^23^. Anxiety and stress were highly associated with smartphone addiction, followed by depression and sleep quality. The results revealed that the likelihood of anxiety and stress is high in smartphone addicts. Selçuk and Ayhan stated that although the risk of smartphone addiction is not related to sleep duration, it is positively associated with depression, anxiety, and stress^24^.

### Limitations

The findings obtained as a result of the research have some limitations. The first of these limitations is that the study was designed in a cross-sectional design. In order to obtain more valid evidence regarding the mediation effect and causal effects between concepts, longitudinal or experimental studies are recommended. Another limitation is that the sample group of the study is limited to university students. In order to examine the effects of the relationships between concepts at different age levels or life periods, it is recommended that different sample groups be preferred in future studies. Adolescence is a risky period in terms of smartphone use, in particular. It is observed that adolescents prefer either too much or too little sleep during this period. In this respect, studies on smartphones and sleep quality in adolescent groups can contribute to the literature.

## CONCLUSION AND RECOMMENDATIONS

As a result of this study, it was observed that depression, stress, and anxiety had a partial mediating effect on the relationship between smartphone addiction and sleep quality of university students. In other words, it may be argued that smartphone addiction contributes to increased levels of depression, stress, and anxiety, which subsequently leads to a decline in sleep quality. Therefore, smartphone addiction appears to be a factor that causes sleep and mental health problems. To address this issue, smartphone addiction should be prevented in order to prevent problems such as stress, anxiety, depression, and insomnia in university students. Informing university students about the correct and functional use of the smartphone and explaining its harmful use should be among the practices that prevent addiction.

## Data Availability

The datasets generated and/or analyzed during the current study are available from the corresponding author upon reasonable request.
